# Planning Today for Tomorrow’s Research: Analysis of Factors Influencing Participation in a Pediatric Cancer Research Biorepository

**DOI:** 10.3389/fonc.2017.00324

**Published:** 2018-01-12

**Authors:** Rania M. Labib, Omneya Hassanain, Maram Alaa, Safaa Ahmed, Sherif Abou El-Naga

**Affiliations:** ^1^Research Department, Children’s Cancer Hospital Egypt 57357, Cairo, Egypt

**Keywords:** sociodemographics, pediatric, biorepository, biobank, middle east, bioethics

## Abstract

**Background:**

Biobanks have become a powerful tool that fosters biomedical research. The success of biobanks depends upon people’s perception and willingness to donate their samples for research. This is the first biorepository in Egypt, hence, little is known about the beliefs and attitudes of parents toward participation.

**Aim:**

To investigate the level of willingness of Egyptians to donate samples of their children and themselves for research and the different factors influencing participation.

**Materials and methods:**

A structured questionnaire was designed covering multiple items expected to affect the enrollment decision. This was conducted in-person, and data collected included demographic data, socioeconomic, and educational level. In addition, in the case of refusal, participants were asked about reasons behind their decision.

**Results:**

Only about 3.1% of patients have not been enrolled in the project, and 0.3% have withdrawn. Three demographic factors were found having disparate trends in the decision-making process to participate or not: father’s education (*p* = 0.0001), mother’s education (*p* = 0.0001), and father’s age (*p* = 0.034).

**Conclusion:**

Egyptian parents were willing to donate their samples as well as their children’s samples in our research biorepository. The idea of participation was presented in an interview during which the consent form was explained in a comprehensive transparent way allowing participants the right to refuse or withdraw at any time. Still, different communication approaches are needed with older, more highly educated parents to encourage them to participate.

## Introduction

Mapping the human genome and the advancement of high-throughput technology has attracted more attention toward the importance of establishing and sustaining human biobanks. Recently, the idea of having research biobanks is spreading widely in many countries. Biobanks are presented as an “organic bank account” to safeguard people’s most valuable biomaterial assets (1). A human biobank (also known as a biorepository) is a vault to collect and archive high quality samples from participants, annotated and linked with all the clinical, demographic, and epidemiologic data of participants for advancing biomedical research (2). Even though the collection and storage of human biospecimen for research has been known for decades, a biobank as a structured harmonized core facility for sample collection is a relatively new tool (3). The value of samples in biobanks is determined in part by the quality as well as by their data richness and their annotation in participant’s clinical data (4).

In November 2012, the Children’s Cancer Hospital Egypt (CCHE) launched the first biorepository core facility in Egypt to take a role in the global war against cancer (2). The success of biobanks depends upon people’s willingness to donate their samples for research. The core dilemma of biobanking, as argued, lies in figuring out how to establish an acceptable frame which can fulfill all the regulations and ethical concerns and not hamper the scientific development. To encourage participation, it is crucial to understand the obstacles and challenges that might affect people’s attitudes toward participation (3). Privacy, the return of results, religious beliefs, as well as socioeconomic and educational level are factors that might have an influence on the decision to participate all factors, which present a greater challenge in a developing country.

The objective of the survey is to explore parents’ attitudes toward sharing biological samples for research and assess the different reasons for refusals taking into consideration the impact of demographics, educational, and socioeconomic level of parents.

## Materials and Methods

### Study Subjects

For every child treated at the CCHE-57357, a biobank interviewer meets his parents/legal guardian during their first visit to inform them about the biobank and ask for their permission to participate. In the case of acceptance, parents should sign the informed consent before sample donation. The consent is written in a simple informative native language approved by the hospital Institutional Review Board (IRB). In signing the consent form, parents have the right to accept or refuse their child’s participation or accept participation to donate certain sample types and refuse other sample types. If parents accept their child’s participation, they are further asked if they accept donating a sample of themselves.

### Sample Collection

If parents approve their child’s participation, a blood sample is collected at the time of acceptance from the child and parents if they are available at the time of signing. In case of a legal guardian, samples were only collected from the child. An additional blood sample is collected from the child at different clinical time points if approved at consenting. At the time of surgery, a tissue sample from the pathology department is supplied to the biorepository.

### The Questionnaire

A structured questionnaire was designed by biobank and epidemiology experts to cover multiple items, which are expected to affect the participation rate. The questionnaire was administered face-to-face at the time of consent. Data were collected including demographics (age, residency, education, and socioeconomic level), previous family history of cancer, and family size. In the case of refusal or withdrawal, reasons should be mentioned and documented specifically.

### Ethical Considerations

Participation was voluntary, and the survey purpose was stated. Confidentiality was granted by agreeing that none of the information disclosed would be used for any reasons other than the legitimate purposes of the survey.

### Statistical Analysis

Continuous variables were compared and expressed as mean ± SD or median (range; continuous variables) and 95% CI. Categorical variables were evaluated using the χ^2^ test or two-tailed Fisher Exact test and expressed as a percentage of the group from which they were derived. Two-tailed tests were used to determine statistical significance; a *p*-value of <0.05 was considered significant. The correlations between responses to each factor and the overall willingness to participate were assessed by calculating phi coefficient. Phi coefficient is used to measure the degree of association between the decision to participate in biobanking and demographic characteristics. Logistic regression was used to evaluate how much of the decision to participate in biobanking can be explained by the variables that were found to be statistically different between the participating and the nonparticipating families.

## Results

### Participation Responses

**Figure 1:**
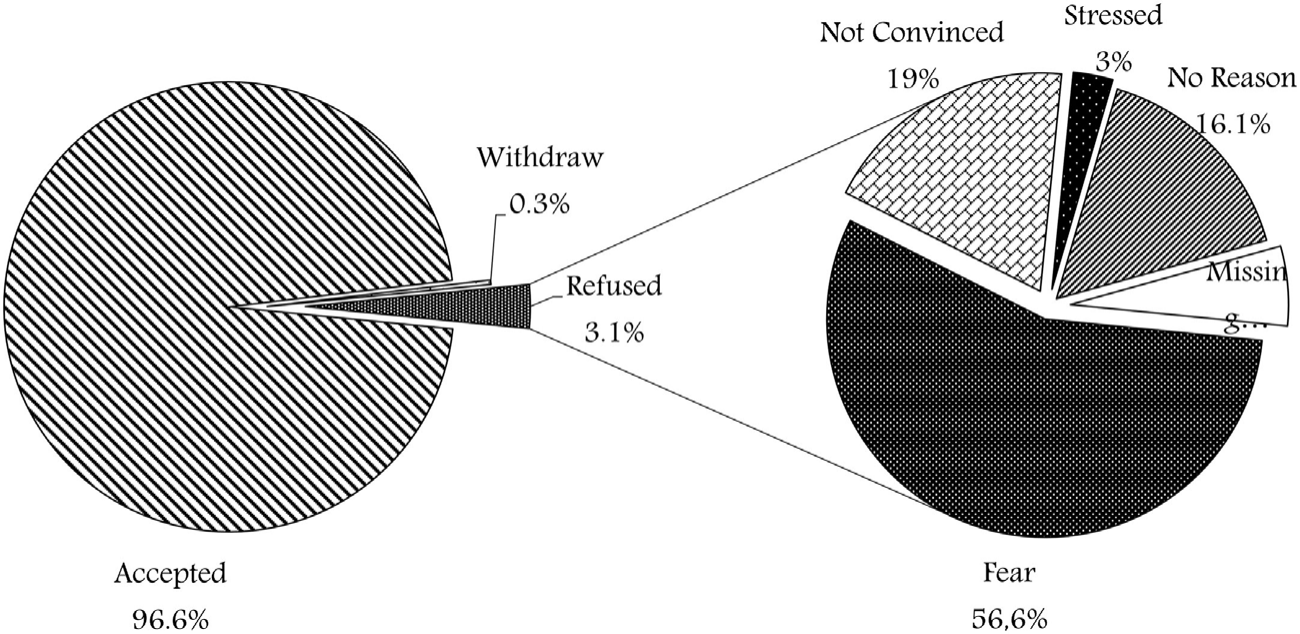
Responses of participant’s parents towards participation in the biobank.

A total of 2,175 participants took part in the survey (35.8% of all participants recruited at the biobank). Figure 1 shows the different responses, 2,100 families (96.6%) were willing to enroll their children and consented, 7 families (0.3%) withdrew after either initial approval or initial sample withdrawal, while only 68 (3.1%) refused to participate. Figure 1 shows the various reasons of refusal, where the majority (56%) of families who refused participation have expressed their fear of the sampling process in view of their current child condition, about 13 (19%) were not convinced with the idea of research and biobanking. Two families (3%) were stressed at the time of consent and expressed that it was inconvenient to take a decision at that time. In addition, 11 families (16%) refused without mentioning any reason. Moreover, four refusal surveys (6%) were missing the reason for non-participation.

### Demographics

Table 1 shows the demographics of parents who took the survey. There was a significant effect of father’s age on the participation decision (*p* = 0.034). The mean age of fathers who accepted was 39.1 ± 0.4 years old while it was 41.3 ± 1.6 years old for those who refused/withdrew. However, there was no significant effect of mothers’ age on the participation decision (*p* = 0.056). The mean age of mothers who accepted participation was 33.1 ± 0.4 years old compared with 34.8 ± 1.7 years old for those who refused/withdrew.

**Table 1:** Parents’ demographic characteristics.

Demographic characteristics	Consented to biobanking (*n* = 2,100)	Refused or withdrew (*n* = 75)	*p*-Value
Father’s age; mean (SD)	39.1 (38.8–39.5)	41.3 (39.6–42.9)	0.034
Mother’s age; mean (SD)	33.1 (32.9–33.5)	34.8 (33.1–36.5)	0.056
**Mother’s level of education**											
Before or finished primary school	468 (22.3%)	7 (9.3%)	0.0001
Finished preparatory, secondary, or technical school	912 (43.4%)	23 (30.7%)	
Education higher than high school	720 (34.3%)	45 (60%)	
**Father’s level of education**											
Before or finished primary school	411 (19.6%)	5 (6.8%)	0.001
Finished preparatory, secondary, or technical school	948 (4.1%)	24 (32.9%)	
Education higher than high school	741 (35.3%)	44 (60.3%)	
**Residency**											
Metropolitan Cairo	818 (39%)	35 (46.7%)	0.5
Lower Egypt and Delta (except coastal governorates)	598 (28.5%)	18 (24%)	
Coastal governorates	289 (13.8%)	11 (14.7%)	
Upper Egypt	255 (12.1%)	5 (6.7%)	
Suez Canal	78 (3.7%)	4 (5.3%)	
Outside Egypt	62 (3%)	2 (4.3%)	
**Population distribution**											
Rural	817 (38.9%)	22 (27.7%)	0.077
Urban	1,283 (61.1%)	53 (70.7%)	

Regarding the effect of education on the participation rate, both the father’s and mother’s level of education had a significant effect on participation (*p* = 0.001 and 0.0001, respectively). We found that those who refused were more likely to have had a higher level of education compared with those who accepted. There was no effect of population rural or urban distribution (*p* = 0.07) nor of location of residency (*p* = 0.5) on the decision of participation.

### Family Size and History of Cancer

Data for the effect of family size and family history of cancer are shown in Table 2. There was no significant difference between sizes of families who accepted to participate compared with those who did not (*p* = 0.99).

**Table 2:** Family size and history of cancer in those who consented to biobanking and those who refused or withdrew their consent.

Family size	Consented (*n* = 2,100)	Refused or withdrew (*n* = 75)	*p*-Value
One child	236 (11.2%)	6 (8%)	0.6
2–4 children	1,645 (78.3%)	62 (82.7%)	
More than 4 children	219 (10.4%)	7 (9.3%)	
**Family history of cancer**	**Consented (*n* = 2,100)**	**Refused or withdrew (*n* = 60)**	***p*-Value**
Yes	817 (38.9%)	14 (29.3%)	0.058
No	1,283 (61.1%)	46 (72.3%)	

Data concerning the family history of cancer were missing for (15/60) families of those who refused/withdrew participation. For the data we have, the family history of cancer had no effect on participation decision (*p* = 0.058).

### Summary of Responses in Relation to Demographics

A logistic regression was performed to evaluate how much of the decision to participate in the biorepository project was influenced by parents’ education; only 4.3% (Nagelkerke *R*^2^) of the variance in consenting was explained by parents’ education. In addition, as explained by the low *R*^2^, all non-consenting was classified as consenting to Biobanking, which is probably due to the low percentage of non-consenting parents and the need for examining more factors that may contribute to the consenting decision.

Three demographic factors were found having disparate trends in the decision-making process to participate or not in the biorepository: father’s education that had a phi coefficient of 0.097 and a significant *p*-value of 0.0001, mother’s education that had a phi coefficient of 0.1 and a significant *p*-value of 0.0001, and father’s age. Parents having a higher education and of older age are more inclined to be unwilling to participate in the biorepository. All the other tested variables had an insignificant *p*-value both for chi-square as well as for the correlation coefficient phi as shown in Tables 1 and 3.

**Table 3:** Correlation of factors influencing decision to participate in biobanking according to correlation with willingness to donate biospecimens for biobanking.

Demographic characteristics	Phi coefficient (*p*)
Mother education	0.1 (0.0001)
Father education	0.097 (0.0001)
Residence	0.043 (0.55)
Population distribution	0.036 (0.094)
Family history of cancer	−0.022 (0.3)

*Phi coefficient was calculated using crosstab procedure in SPSS to measure the degree of association between the decision to participate in biobanking and demographic characteristics*.

## Discussion

One of the factors affecting biobank sustainability is public engagement and beliefs about research (5). In this article, we aim to investigate the pattern of participants’ willingness to donate samples for research and the various reasons affecting their decision. Our biobank is the first biobank in Egypt, and this study is to investigate the willingness of Egyptians to donate samples for research with biological samples donation on approval. The special condition that we collect samples from children as well as from parents represented an added challenge.

Developing countries should shape the communities’ bioethics beliefs to step into the post-genomic era. In a balanced community, the moral attributes dictate that the concept of citizenship includes an individual’s awareness of rights and duties of the self and its relation with others (6). Although we have made it clear in the interview process that it is unlikely for participants to have any direct benefit themselves, about 97% of interviewed parents accepted to participate. The majority of parents have expressed that their driving motive was the desire to help discover the mystery of cancer, find cures, and alleviate the pain of children. In agreement with our findings, a recent study showed that people tend to participate in research if it is aiming at preventing or finding a cure for the disease they are suffering from (7). In Sweden, a similar survey was done and showed that about 86% agreed to potentially donate a blood sample for research purposes and a total of 78% agreed to donate and store their samples and that was derived by emotional motives (8).

Our study found that parents who refused to participate in the biobank were fathers of older age rather than those of older age (*p* = 0.034) whereas mother’s age was of no significant influence (*p* = 0.056). This was in agreement with another study in Jordan where individuals aged 60 years or older had a negative attitude regarding participation in a biobank (*p* < 0.001) (9). By contrast, a previous study mentioned that older age was a strong dependent factor for participation where the highest participation (85.6%) was among the older group (60–69 years old) compared with 49% acceptance for those aged 20–29 years old (10).

Parents’ level of education is another important factor reflecting parents’ culture and beliefs while taking the decision regarding participation. In this study, analyzing the data of parents who refused participation showed that the refusal rate was mainly among parents who were educated above high school compared with those who were illiterate or educated below the level of high school. In agreement with our results, people with higher education had a more restrictive attitude toward research and were willing to be informed about the purpose of specific research requesting samples and not an open consent for biobanking (11). In contrast, Ahram et al. showed that willingness to donate samples for research was higher as the participants’ level of education increased (9). Moreover, an Australian cohort study showed that people with a tertiary education in a regional area were significantly more likely to participate than those without a tertiary education (12). In addition, a study by Gaskell et al. stated that the donation of samples was associated with those of a higher level of education (*r* = 0.097, *p* < 0.001) (13). Whereas, an Italian survey did not identify education as an independent factor affecting the participation decision and concluded that participation or not is an individual feeling or belief affecting his choice, i.e., something not necessarily related to formal education or scientific knowledge (3).

Another studied factor was the geographical distribution of participants and how it may influence the recruitment plan. In a previous study, participation varied significantly between the urban and regional areas (12); however, in our study, it had no effect on the decision of participation.

In the biobanking community, privacy and protection of participants’ information is one of the major issues affecting individual participation. Privacy is maintained by removing any data that might lead to participant identification such as name, medical record number, address, date of birth, phone, fax, and email (14). Those de-identified, coded samples are made available for release to different investigators according to the policy of the institution and after IRB approval. In our case, fear concerning data protection or patient identification was not causing any level of anxiety for any of the participants, and we attribute this to the lack of people’s awareness concerning their privacy rights. In this survey, we have not asked for any financial information. This was in agreement with a study by Gaskell et al. who had shown that individuals who were willing to participate had fewer concerns about privacy (13). Nevertheless, a study by Kaufman et al. showed that 88% of individuals were concerned about the privacy of their financial information, and 79% were concerned about the privacy of their medical information (14). In Sweden, privacy was found to be the main cause of people’s refusal to participate in a biobank (15) while participants in an Italian survey by Porteri et al. had expressed that they preferred that the de-identification and coding of samples is made with the possibility to be identified later if needed (97%) rather than the complete anonymization of samples, which does not allow any future identification of donors (3).

The return of research results is a debatable issue in biobanks where some researchers find an ethical obligation to return genetic data results to participants. In our consent, we mention that we are not going to return results to participants; however, we state that in some cases, we might return results to the primary physician. Our policy of not returning research results to participants had no impact on participation rate as it was not mentioned as a concern in any refusal cases. Different researchers tried to assess participant’s attitudes toward these issues and found that some participants were very eager to receive their results (16). Also, Glass et al. agreed that the return of research results increases the willingness of participation in any type of research (7).

In addition, research beliefs were important because they shape individual’s formal and informal norms. Religion builds the basis for what is considered right and wrong; therefore, there is a strong link between religion and sample donation, volunteering and the concept of helping others get the best cure in the future. Some religious misconcepts and beliefs may potentiate people’s fear to donate their body pieces as this may interfere with God’s will or have concepts about importance of burying their body parts. In our survey, none of the participants showed that the refusal was based on any religious reason and we have not experienced any religious barriers hindering Egyptians from participating in research with samples or data. As such, we did not find a need to analyze people’s religion as a factor. The study of Ahram et al. showed that participation was positively influenced by religious inclination for both Muslims and Christians (9).

### Limitations of Our Study

Our biobank is the first biobank in Egypt and during the establishment phase, we did not have an idea about people’s attitude and the rate of acceptance or refusal so we designed a survey to guide us to different reasons that might affect people’s participation.

Our cohort was parents at their initial visit to CCHE who were very emotional, shocked, and confused by the knowledge that their child has cancer. This cohort might not represent the normal population.

Return of research results is a hot topic within the biobanking community as a way to benefit people who participated. A study by Lemke et al. conducted a survey on the willingness of participants to have their research results back, and they found that over 97% of participants were willing to have their research results back (17). However, this item was not analyzed in our setting as we do not provide data back to participants although some parents had inquiries about the return of research results, but we could not promise them that we can offer the result except if something critical was found.

## Conclusion

Overall, our study presents insights into different attitudes toward participation in research. We believe that each biobank should understand the pattern of its population to design the materials that facilitate comprehending the idea of the project and answer all participants’ queries. Moreover, there should be more training designed for the interviewers to present the project in multiple and better ways according to the level of participants’ understanding.

## Ethics Statement

All subjects gave written informed consent in accordance with the Declaration of Helsinki and was approved by “Children’s Cancer Hospital Egypt-57357-IRB”.

## Author Note

Location of study: Children’s Cancer Hospital Egypt-57357. IRB approval for patient study: Children’s Cancer Hospital Egypt-57357.

## Author Contributions

RL designed study and wrote the manuscript. OH designed the survey and analyzed data. MA and SA collected data and helped in writing the manuscript. SE-N revised the manuscript.

## Conflict of Interest Statement

The authors declare that the research was conducted in the absence of any commercial or financial relationships that could be construed as a potential conflict of interest.

## References

[1] Alice P 10 Ideas Changing the World Right Now: Biobanks. Available from: http://content.time.com/time/specials/packages/article/0,28804,1884779_1884782_1884766,00.html

[2] Labib RM Mostafa MM Alfaar AS Yehia D Alaa M Elzayat MG Biorepository for pediatric cancer with minimal resources: meeting the challenges. Biopreserv Biobank (2016) 14(1):9–16. 10.1089/bio.2015.0004 26691960

[3] Porteri C Pasqualetti P Togni E Parker M Public’s attitudes on participation in a biobank for research: an Italian survey. BMC Med Ethics (2014) 15(1):81 10.1186/1472-6939-15-81 25425352 PMC4258254

[4] Ormond KE Cirino AL Helenowski IB Chisholm RL Wolf WA. Assessing the understanding of biobank participants. Am J Med Genet A (2009) 149A(2):188–98. 10.1002/ajmg.a.32635 19161150

[5] Watson PH Nussbeck SY Carter C O’Donoghue S Cheah S Matzke LAMM A framework for biobank sustainability. Biopreserv Biobank (2014) 12(1):60–8. 10.1089/bio.2013.0064 24620771 PMC4150367

[6] Rose N Novas C Biological Citizenship. In: Ong A Collier SJ, editors. Global Assemblages: Technology, Politics, and Ethics as Anthropological Problems. Oxford: Blackwell Publishing (2005). p. 439–63.

[7] Glass DC Kelsall HL Slegers C Forbes AB Loff B Zion D A telephone survey of factors affecting willingness to participate in health research surveys. BMC Public Health (2015) 15:1017. 10.1186/s12889-015-2350-9 26438148 PMC4594742

[8] Kettis-Lindblad A Ring L Viberth E Hansson MG. Genetic research and donation of tissue samples to biobanks. What do potential sample donors in the Swedish general public think? Eur J Public Health (2006) 16(4):433–40. 10.1093/eurpub/cki198 16207726

[9] Ahram M Othman A Shahrouri M Mustafa E. Factors influencing public participation in biobanking. Eur J Hum Genet (2014) 22(4):445–51. 10.1038/ejhg.2013.174 23921537 PMC3953902

[10] Holmen J Midthjell K Krüger Ø Langhammer A Holmen TL Bratberg GH The Nord-Trøndelag Health Study 1995–97 (HUNT 2): objectives, contents, methods and participation. Nor Epidemiol (2003) 13(1):19–32.

[11] Nilstun T Hermerén G Human tissue samples and ethics. Med Health Care Philos (2006) 9(1):81–6. 10.1007/s11019-005-7984-4 16645800

[12] Banks E Herbert N Mather T Rogers K Jorm L Characteristics of Australian cohort study participants who do and do not take up an additional invitation to join a long-term biobank: the 45 and Up Study. BMC Res Notes (2012) 5:655 10.1186/1756-0500-5-655 23181586 PMC3536556

[13] Gaskell G Gottweis H Starkbaum J Gerber MM Broerse J Gottweis U Publics and biobanks: Pan-European diversity and the challenge of responsible innovation. Eur J Hum Genet (2013) 21(1):14–20. 10.1038/ejhg.2012.104 22669414 PMC3522201

[14] Kaufman DJ Murphy-Bollinger J Scott J Hudson KL. Public opinion about the importance of privacy in biobank research. Am J Hum Genet (2009) 85(5):643–54. 10.1016/j.ajhg.2009.10.002 19878915 PMC2775831

[15] Johnsson L Hansson MG Eriksson S Helgesson G Patients’ refusal to consent to storage and use of samples in Swedish biobanks: cross sectional study. BMJ (2008) 337:a345 10.1136/bmj.a345 18617496 PMC2656925

[16] Johnsson L Helgesson G Rafnar T Halldorsdottir I Chia K-S Eriksson S Hypothetical and factual willingness to participate in biobank research. Eur J Hum Genet (2010) 18(11):1261–4. 10.1038/ejhg.2010.106 20648060 PMC2987483

[17] Lemke AA Halverson C Ross LF. Biobank participation and returning research results: perspectives from a deliberative engagement in South Side Chicago. Am J Med Genet A (2012) 158A(5):1029–37. 10.1002/ajmg.a.34414 22438108 PMC3331902

